# Study on the Similarity of Biomechanical Behavior between Gelatin and Porcine Liver

**DOI:** 10.1155/2020/7021636

**Published:** 2020-08-22

**Authors:** Jiyun Zhao, Chao Cao, Guilin Li, Liuyin Chao, Haigang Ding, Yufeng Yao, Liangchen Song, Xin Jin

**Affiliations:** ^1^School of Mechatronic Engineering, China University of Mining & Technology, Xuzhou 221116, China; ^2^Jiangsu Key Laboratory of Mine Mechanical and Electrical Equipment, China University of Mining & Technology, Xuzhou 221116, China; ^3^Xuzhou Maternal and Child Health Care Hospital, Xuzhou 221000, China; ^4^Department of Engineering Design and Mathematics, University of the West of England, Coldharbour Lane, Bristol BS16 1QY, UK

## Abstract

As a natural polymer, gelatin is increasingly being used as a substitute for animals or humans for the simulation and testing of surgical procedures. In the current study, the similarity verification was neglected and a 10 wt.% or 20 wt.% gelatin sample was used directly. To compare the mechanical similarities between gelatin and biological tissues, different concentrations of gelatin samples were subjected to tensile, compression, and indentation tests and compared with porcine liver tissue. The loading rate in the three tests fully considered the surgical application conditions; notably, a loading speed up to 12 mm/s was applied in the indentation testing, the tensile test was performed at a speed of 1 mm/s until fracture, and the compression tests were compressed at a rate of 0.16 mm/s and 1 mm/s. A comparison of the results shows that the mechanical behaviors of low-concentration gelatin samples involved in the study are similar to the mechanical behavior of porcine liver tissue. The results of the gelatin material were mathematically expressed by the Mooney-Rivlin model and the Prony series. The results show that the material properties of gelatin can mimic the range of mechanical characteristics of porcine liver, and gelatin can be used as a matrix to further improve the similarity between substitute materials and biological tissues.

## 1. Introduction

The range and importance of material characteristics, such as biodegradability, biocompatibility, and mechanical behavior, which need to be addressed vary depending on the application. As far as biomechanical properties are concerned, it is generally believed that the mechanical behavior of biomaterials in different environments and different parts of the tissue is significantly different at different loading speeds [1]. Taking porcine liver as an example, Samur et al. [1] measured the in vivo liver stress release curve within one hour after pig death under different compression speeds ranging from 0.5 mm/s to 8 mm/s. Gao and Desai [2] performed mechanical measurements on thawed porcine liver tissue at a tensile speed of 1.27 mm/s. Chui et al. [3] measured the stress-strain curve of porcine livers at a maximum speed of 3.33 mm/s. The above curves all showed differences in the mechanical behavior of porcine liver tissue, especially when the deformation is large.

At the same time, due to the availability of materials, ease of preparation, and ethical significance, gelatin is increasingly replacing natural biological tissues for research applications [4, 5] in medical engineering, including forensic and military wound profiling and projectile damage simulation objects [6, 7], medical phantom materials in imaging [8, 9], tissue regeneration materials [10, 11], and tissue substitutes in surgical simulations [12]. However, previous research has focused on the application of gelatin in high-speed projectile damage simulation [13]. Recently, the time and rate dependence of the gelatin mechanical response was studied by Ravikumar et al. [14] at speeds of 0.1 to 1 mm/s, but the speed range was not sufficient to cover surgical applications, especially as the latest medical jet studies expanded their speed by several orders of magnitude [14–16]. Current research also lacks a comparison of the similarities with biological tissue.

The above analysis indicates that in order to expand the surgical application of gelatin, a large amount of testing and data analysis work is required to study the difference in the mechanical similarity between gelatin and specific biological tissues [17]. In addition, an interesting phenomenon is that current surgical simulated gelatin is configured with a mass fraction of 10 wt.% or 20 wt.% [14–16], in which relevant proof studies are lacking. This study compares the mechanical response of gelatin to specific biological tissues at different loading speeds and summarizes the gelatin configuration method with a high similarity to soft tissue mechanical behavior.

## 2. Materials and Methods

To comprehensively compare the differences in the mechanical response between porcine liver and gelatin, uniaxial stretching, compression, and indentation tests were carried out in sequence. The concentration differences of gelatin samples are considered during the configuration while ensuring consistent sample preparation environments. The differences in the different parts in the preparation of the samples were considered when preparing porcine liver samples.

### 2.1. Sample Preparation

According to the current research, gelatin concentrations of 10 wt.% to 20 wt.% are used as a reference, and the gelatin concentration is appropriately decreased based on the low hardness exhibited by the liver tissue compared with other soft tissues. However, low-concentration gelatin is difficult to form and is prone to breakage during demolding, resulting in samples that do not meet the requirements. Therefore, in order to fully consider the mechanical response of different concentrations of gelatin, the concentration of gelatin samples in this study ranged from 8 wt.% to 20 wt.% with 2 wt.% intervals. The pig skin-extracted Sigma 48722 gelatin powder was dissolved in 50°C distilled water and stirred at a constant temperature for 1 hour. After the bubbles disappeared, it was poured into a mold and solidified at a constant temperature of 6°C for 24 hours. Thus, a strip sample having a length of 90 mm, width of 10 mm, and thickness of 10 mm for the tensile test; a cylindrical sample with a height of 10 mm and diameter of 10 mm for the compression test; and a cylindrical sample with a height of 50 mm and diameter of 40 mm for the indentation test were prepared. Cracks and breakage occurred in the gelatin sample during the demolding process. In this study, multiple sets of samples with various shapes were prepared for different concentrations. After demolding, the samples with a good shape and size were selected to perform the experiment. Porcine liver tissue obtained from a slaughterhouse within two hours after death was used for the sample preparation, and three kinds of samples with the same size as the gelatin samples were prepared separately from the previous mold. To account for the differences in different parts of the liver tissue, four porcine liver samples for compression and stretching were selected from different parts. Three six-month-old sows were removed from the slaughterhouse within two hours of slaughter. A sample of the same size as the gelatin sample was prepared. Special care should be taken when selecting tissue specimens. The sample was extracted perpendicular to the upper surface of the liver. The outer liver membrane (capsule) was excised. In experiments, samples with large blood vessels or large holes were discarded to test the properties of tissue in essentially the same areas. Because the required sample thickness is greater than that of some parts of the liver, the sample locations on the pig liver are relatively concentrated.

### 2.2. Measurement Methods

**Figure 1:**
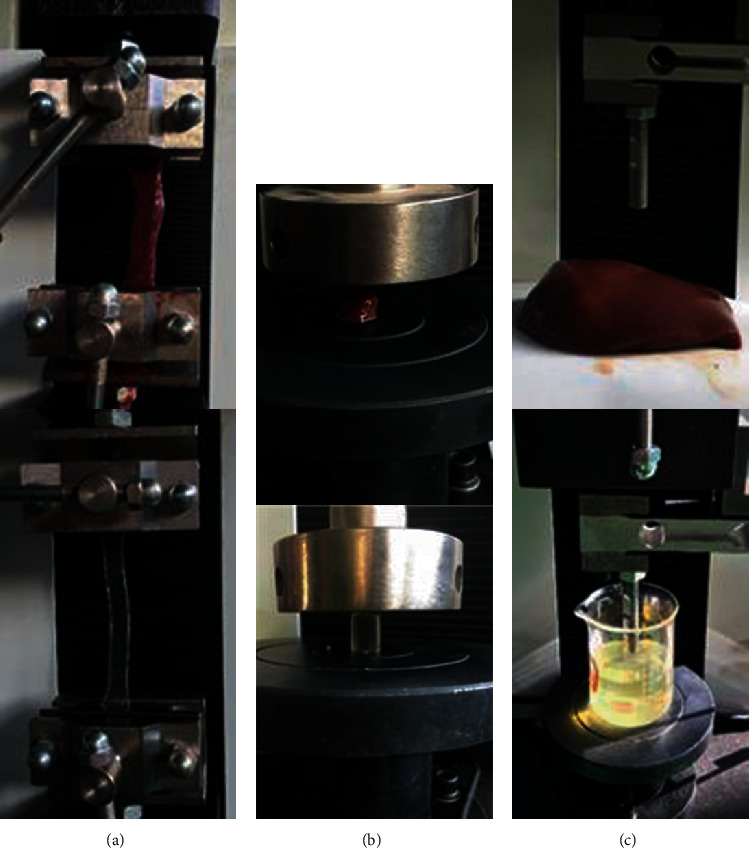
Experimental process: (a) tensile testing; (b) compression testing; (c) indentation testing.

The test machine provides a moving speed of 0.01 to 13.3 mm/s, and the Celtron LPS sensor on the test machine has a force range of 0 to 200 N. The clamps or indenter on the test machine can be replaced to clamp or contact the sample for loading. As shown in Figure 1, the upper and lower stretching clamps are 20 mm in height to ensure that the length of the clamped sample is 50 mm. A disk-shaped indenter with a diameter of 100 mm is used for compressing the sample, and a rod-shaped indenter with a diameter of 10 mm is used for the indentation test. In the compression and indentation tests, software is used to measure the displacement of the closed loop, which ensures that the computer starts to record data after the indenter is in contact with the sample, and the bottom surface is kept flat when the indenter is installed.

In the three tests, corresponding to all test cases, the test results of the gelatin samples are reflected by the average value obtained through the same three measurements. In fact, the results of the tensile test and the compression test are both damaged, and the deformation no longer recovers. For the porcine liver samples, due to the different sampling location of the liver, the mechanical expression results of porcine liver samples often vary greatly. Under the above circumstances, we believe that the results are credible only when the test results of porcine liver samples are repeatable; there were more test samples than valid data samples. Therefore, corresponding to the tensile and compression tests of porcine liver samples, 6 samples were prepared to reflect the repeatable results. For the indentation test, neither gelatin nor porcine liver samples formed fracture failure, so three repetitive experiments were carried out under each loading condition, and the average value was used to express the test results.

## 3. Results

### 3.1. Tensile Testing

**Figure 2:**
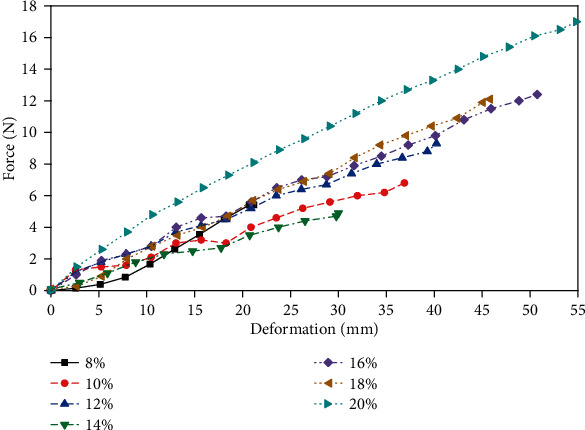
Tensile deformation-force relationship of gelatin.

By keeping the lower stretching clamp fixed, the upper clamp moves upward at a constant speed of 1 mm/s until the specimen breaks.  Figure 2 compares the deformation-force differences between different concentrations of gelatin. The relationship between deformation and force of the gelatin is basically linear, and the greater the concentration is, the greater the value of deformation at failure. The 8 wt.% gelatin sample failed when the force rose to 5.42 N, which was the smallest fracture deformation in all samples, and the deformation was 21.17 mm. Another general trend is that the lower the concentration is, the lower the stress of gelatin under the same deformation amount, but this trend is not obvious when the deformation is larger than 20 mm.

**Figure 3:**
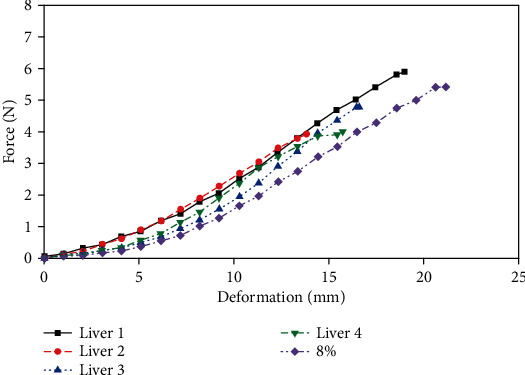
Tensile deformation-force comparison between 8 wt.% gelatin and porcine liver.

After the tensile testing of 4 groups of porcine liver tissues, the tensile deformation-force relationship between porcine liver and gelatin was compared, and the tensile deformation-force curve of gelatin with a concentration of 8 wt.%, which is closest to that of porcine liver tissue, was used as a reference in Figure 3. The results showed that the tensile-deformation curves of porcine liver samples have a good consistency, and the degree of nonlinearity exhibited by low tensile deformation was greater than that of gelatin. At the same time, the fracture deformation value of porcine liver tissue was smaller than that of gelatin. The tensile stress of the porcine liver tissue and the gelatin sample with a concentration of 8 wt.% to 16 wt.% in the deformation range of 20 mm was similar.

### 3.2. Compression Testing

The sample was placed at the center of the horizontal chassis of the testing machine, and the underside of the indenter was horizontally mounted. To minimize the influence on the testing from the friction and adhesion between the sample and the chassis or indenter, oil is applied between the contact surfaces for lubrication. The two groups were compressed at a rate of 0.16 mm/s and 1 mm/s, respectively. Each group included two different samples from sections of porcine liver and gelatin samples with concentrations ranging from 8 wt.% to 20 wt.%. The test data for each set of samples are recorded from when the indenter contacts the sample to when the force peak occurs.

**Figure 4:**
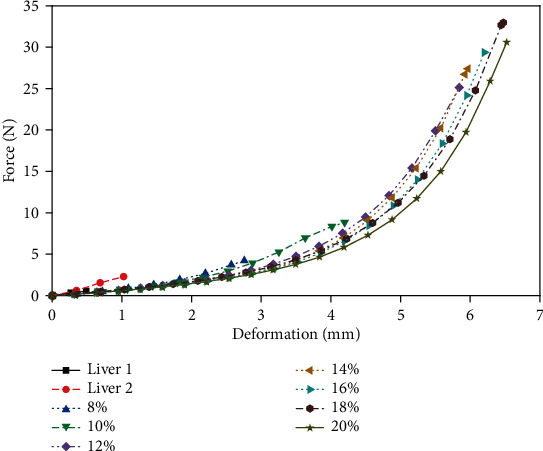
Force-deformation curve of porcine liver and gelatin at a 0.16 mm/s compression rate.

**Figure 5:**
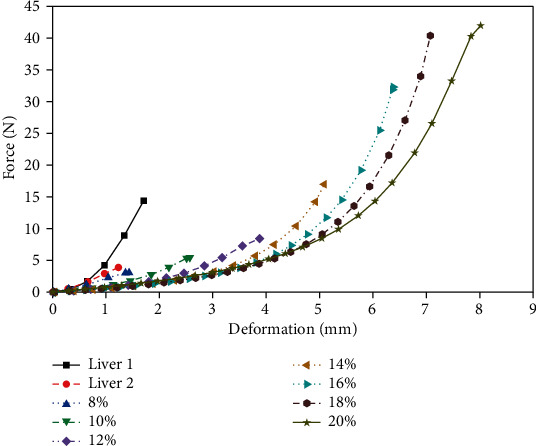
Force-deformation curve of porcine liver and gelatin at a 1 mm/s compression rate.

Figure 4 shows the relationship between deformation and compressive stress in the porcine liver and gelatin samples at a speed of 0.16 mm/s. The difference in the maximum deformation between the two porcine liver samples was significant. The maximum compressive deformation of the gelatin samples is much larger than that of porcine liver tissue, and the higher the gelatin concentration is, the greater the value of deformation. At the same time, under the same deformation, the compressive stress of gelatin samples with a concentration of 8 wt.% to 20 wt.% is smaller than that of porcine liver tissue. The gelatin sample showed a large nonlinear relationship between the force and the deformation after the compression exceeded 3 mm. With the increase in the concentration, the compressive stress of the gelatin sample decreased under the same deformation. Similarly, Figure 5 compares the relationship between deformation and compressive stress in the porcine liver and gelatin samples at a speed of 1 mm/s. In the case of a higher compression rate, although the curve change trend remains unchanged, the maximum deformation and the maximum force of the two materials are increased, and the difference between the samples is more significant.

It can be seen from the comparison of Figures 4 and 5 that in the compression experiment, the high-speed loading can increase the maximum deformation of the two materials and thereby increase the maximum compressive stress. The largest difference between the gelatin and porcine liver is in the range of force-deformation; the gelatin material can withstand a large compressive stress. Within the range of gelatin concentrations in this study, low concentrations of gelatin, especially 8 wt.%, were closer to porcine liver tissue in terms of compressive force response.

### 3.3. Indentation Testing

Indentation experiments were performed on porcine liver and gelatin with the same indenter. The force values within 60 seconds after the indenter came in contact with the surface of the sample were recorded. First, the porcine liver samples were compressed for 1 second at different indenter movement speeds of 4 to 12 mm/s. To compare the stress release process at different head speeds with the same compression displacements, the samples were compressed at 4 and 12 mm/s for 4 mm and 12 mm, respectively. Second, in order to capture the difference between the porcine liver and samples with different concentrations of gelatin, the stress release process after each sample which was compressed to 10 mm at a rate of 10 mm/s was tested. Finally, the same test as the porcine liver sample was performed for the gelatin samples with a concentration of 10 wt.% for comparison.

**Figure 6:**
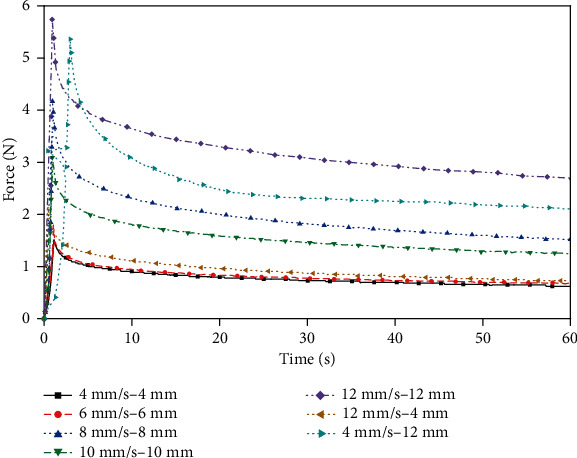
Porcine liver indentation force-time relationship.

**Figure 7:**
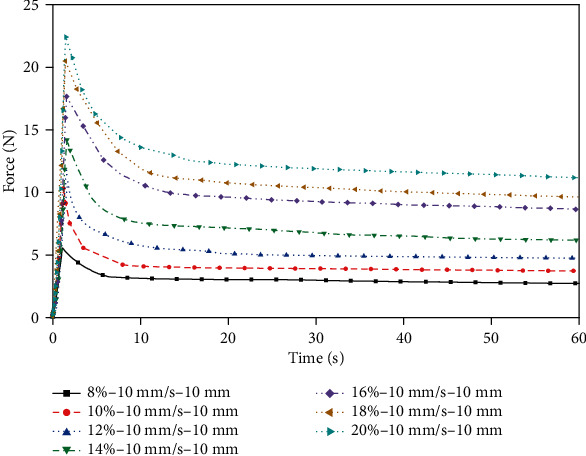
Indentation force-time relationship of different concentrations of gelatin.

**Figure 8:**
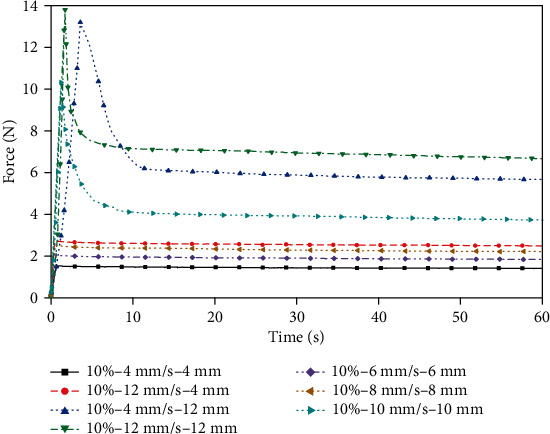
The 10 wt.% concentration gelatin indentation force-time relationship.

Figure 6 shows that when the porcine liver is compressed at a higher speed for 1 second, the larger the deformation of the sample is, the greater the elastic energy stored in the sample, the larger the force peak, and the longer the stress release time. The above differences were small when the porcine liver was compressed at speeds of 4 mm/s and 6 mm/s. Comparing the curves of 4 mm and 12 mm at 4 mm/s or 12 mm/s, it can be seen that the higher the compressing speed is, the higher the peak force when compressed to the same depth.  Figure 7 shows that the higher the gelatin concentration is, the greater the force peak. According to the comparison of Figures 6 and 7, the stress release of the gelatin sample is more gradual. Judging from the peak of the force, 8 wt.% gelatin is closer to the mechanical response behavior of the porcine liver under this loading condition.  Figure 8 shows the force-time curve of the 10 wt.% gelatin sample. The mechanical response, which is reflected as a relationship of the compression speed and compression displacement, is the same as that of porcine liver, as follows: for the same loading time, the higher the compression speed is, the greater the force peak; for the same compression depth, the higher the compressing speed is, the greater the force peak.

**Figure 9:**
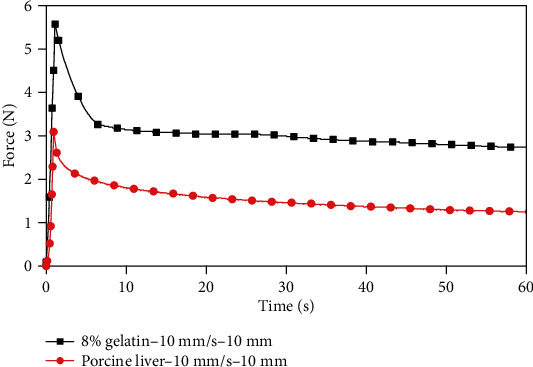
Indentation force-time relationship between the 8 wt.% concentration gelatin and porcine liver.

Figure 9 shows the force-time response relationship between 8% gelatin and porcine liver at a compression rate of 10 mm/s to 10 mm. It can be seen from the figure that 8% gelatin and porcine liver have a biomechanical consistency, and the mechanical response relationship between them differs little. In the force-time response curve, the stress release trend of 8% gelatin and porcine liver was completely consistent, and the stress peak value difference of 8% gelatin and porcine liver was only 2.48 N, which was very small compared with other gelatin samples with other concentrations. Therefore, 8% gelatin and porcine liver have a high degree of biomechanical similarity under this loading condition.

## 4. Discussion

The above experimental results show that although the tensile behavior response of the gelatin material in the small deformation range and the compression response under high-speed loading are different from those of the porcine liver tissue, the parameters of the mechanical behavior of the gelatin material can mimic the range of the porcine liver material. This shows that the improved configuration method with gelatin as the ground substance matrix would be closer to the mechanical properties of biological tissues.

Among the ranges of gelatin concentration involved in this study, low concentrations, especially 8% gelatin samples, have a greater similarity to pig liver. Further, in order to clarify the hyperelastic and viscoelastic mechanical behavior of gelatin materials, this study calculated the constitutive model parameters of the samples.

### 4.1. Hyperelastic Model

A well-known approach for studying nonlinear constitutive relationships of bodies capable of finite deformation is to postulate that elasticity has the form of an elastic potential or strain energy function, *W*. The strain energy for an elastic body is a function of the state of deformation.

For isotropic, hyperelastic, and incompressible materials, the strain energy equation is written as

(1) $$\begin{array}{c} W=F_{1}\left(I_{1},I_{2}\right)+F_{2}\left(\lambda \right)+F_{3}\left(I_{1},I_{2},\lambda \right), \end{array}$$

where *I*_1_ and *I*_2_ are the invariants of the right Cauchy stretch tensor and *λ* is the stretch along the stretching direction. The function *F*_1_ represents the behavior of the ground substance matrix, while *F*_2_ represents the behavior of the fibers or fascia, and *F*_3_ represents an interaction between the matrix and the fibers that are presumed to take the form of a shear coupling. For uniaxial testing, there is no shear in the matrix with respect to the fibers; thus, the *F*_3_ component is not included. According to the most common Mooney-Rivlin material model based on the modeling of the mechanical behavior of porcine liver tissue [2, 3, 18, 19],

(2) $$\begin{array}{c} F_{1}=\frac{C_{1}}{2}\left(I_{1}-3\right)+\frac{C_{2}}{2}\left(I_{2}-3\right), \end{array}$$

where *C*_1_ and *C*_2_ represent the Mooney-Rivlin coefficients.

The fibers or fascia is assumed to be unable to resist compressive loading; thus, the model is isotropic. An exponential function describes the straightening of the fibers or fascia, while a linear function describes the behavior of the fibers or fascia once it is straightened past a critical stretch level [20], namely,

(3) $$\begin{array}{c} \frac{\partial F_{2}}{\partial \lambda }=0,\;\lambda <1, \end{array}$$

(4) $$\begin{array}{c} \frac{\partial F_{2}}{\partial \lambda }=\frac{C_{3}}{\lambda }\left(e^{C_{4}\left(\lambda -1\right)}-1\right),\;\lambda <\lambda^{*}, \end{array}$$

(5) $$\begin{array}{c} \frac{\partial F_{2}}{\partial \lambda }=\frac{1}{\lambda }\left(C_{5}\lambda +C_{6}\right),\;\lambda \geq \lambda^{*}, \end{array}$$

where *C*_3_, *C*_4_, *C*_5_, and *C*_6_ represent coefficients, and the value of *C*_6_ depends on *C*_3_, *C*_4_, *C*_5_, and *λ*^∗^, to ensure that *F*_2_ is continuous.

Let **X** denote a point in the reference configuration. The current position of the point is denoted by *x*, where *x* is a function of time. The gradient of *x* with respect to **X** is called the deformation gradient

(6) $$\begin{array}{c} F={\left(\frac{\partial x}{\partial X}\right)}^{T}. \end{array}$$

The Cauchy stress, *σ*, is related to **S** by

(7) $$\begin{array}{c} \sigma =\frac{1}{J}F\cdot S\cdot F^{T}, \end{array}$$

where *J* = det **F**. For incompressible materials, *J* = 1.

For incompressible materials, the Cauchy stress in the direction of stretching can be written as the partial derivative of the strain energy function *W*.

(8) $$\begin{array}{c} \sigma =2\frac{\partial W}{\partial I_{1}}\left(\lambda^{2}-\frac{1}{\lambda }\right)+2\frac{\partial W}{\partial I_{2}}\left(\lambda -\frac{1}{\lambda^{2}}\right). \end{array}$$

As the Cauchy stress, *σ*, is related to the first Piola-Kirchhoff stress tensor, *T*

(9) $$\begin{array}{c} \sigma =\lambda T. \end{array}$$

From (8),

(10) $$\begin{array}{c} T=\frac{2}{\lambda }\frac{\partial W}{\partial I_{1}}\left(\lambda^{2}-\frac{1}{\lambda }\right)+\frac{2}{\lambda }\frac{\partial W}{\partial I_{2}}\left(\lambda -\frac{1}{\lambda^{2}}\right). \end{array}$$

If the original cross-sectional area of the sample used in the experiment is *A*_0_, the tensile or compressive load is *F*, the original length of the sample is *L*_0_, and the extension length is *L*, then

(11) $$\begin{array}{c} T=\frac{F}{A_{0}}, \end{array}$$

(12) $$\begin{array}{c} \lambda =\frac{L_{0}+L}{L_{0}}. \end{array}$$

The data of the tensile test of the 8 wt.% gelatin sample were taken, and the parameters were fitted by the least squares method according to Equations (10) through (12). Considering the behavior of fibers and fascia, the results are as follows: *C*_1_ = 0.05769 MPa, *C*_2_ = −0.0641 MPa, *C*_3_ = 0.001307 MPa, *C*_4_ = 29.73, *C*_5_ = 0.0133 MPa, and *λ*^∗^ = 1.05, and the variance of the experimental data and fitting results is *R*^2^ = 0.9985. If gelatin is regarded as a simple matrix, *C*_1_ = 0.05587 MPa, *C*_2_ = −0.06022 MPa, and *R*^2^ = 0.9982. It can be seen that *C*_1_ and *C*_2_ have little difference among the above two parameters, and the deviations are 3.15% and 6.05%, respectively. Similarly, the data of the compression test of the 8 wt.% gelatin sample were taken, and the parameters were fitted by the least squares method according to Equations (10) through (12). Considering the behavior of fibers and fascia, the results are as follows: *C*_1_ = 0.76728 MPa, *C*_2_ = −0.6372 MPa, *C*_3_ = 0.02614 MPa, *C*_4_ = 21.34, *C*_5_ = 0.0798 MPa, and *λ*^∗^ = 1.05, and the variance of the experimental data and fitting results is *R*^2^ = 0.9786. Therefore, a gelatin sample can be used to replace porcine liver tissue for mechanical response under the above-described loading conditions.

### 4.2. Viscoelastic Model

The stress release data from the 8 wt.% gelatin sample compressed at 10 mm/s for 10 mm was calculated using a Prony series.

The relaxation modulus is *E*(*t*), and the transient modulus is *E*_0_.

(13) $$\begin{array}{c} E\left(t\right)=E_{0}\left[1-\overset{n}{\underset{i=1}{\sum }}s_{i}\left(1-e^{-t/\tau_{1}}\right)\right], \end{array}$$

where *s*_*i*_ is the coefficient and *τ*_*i*_ is the time constant corresponding to every stage.

As the force *F*(*t*) is related to the relaxation modulus *E*(*t*),

(14) $$\begin{array}{c} F\left(t\right)=\frac{2rh}{\left(1-v^{2}\right)}E\left(t\right), \end{array}$$

where *r* is the radius of the indenter and *h* is the depth of the indentation. *ν* is the material Poisson's ratio.

This study uses a 6-order Prony series, i.e., *n* = 6, and Equations (13) and (14) are written as

(15) $$\begin{array}{c} F\left(t\right)=\frac{2rh}{\left(1-v^{2}\right)}E_{0}\left[1-\overset{6}{\underset{i=1}{\sum }}s_{i}\left(1-e^{-t/\tau_{1}}\right)\right]. \end{array}$$

The stress relaxation fitting results characterizing the viscoelasticity are shown in Table 1.

The variance between the above fitting results and the experimental data was 0.9509, indicating that the gelatin sample conformed to the expression of the viscoelastic behavior under the experimental loading conditions.

## 5. Conclusions

In this study, the mechanical behaviors of porcine liver tissue and gelatin samples under different loading conditions were analyzed by tensile, compression, and indentation experiments. Although the mechanical responses of gelatin samples and porcine liver tissue are different in some experiments, the material properties of gelatin could mimic the range of mechanical characteristics of porcine liver. The gelatin sample with a concentration of 8% is closer to the mechanical expression of porcine liver, especially at the deformation rate of 10 mm/s. The constitutive parameters of the gelatin material change little regardless of whether the effect of the fiber or fascia is considered. The results of the two hyperelastic constitutive models of gelatin indicate that it is feasible to further update the configuration process or add materials with gelatin as a matrix to improve the similarity between the materials and biological tissues.

For liver tissue, under large strains, stress with strain increases almost linearly in most existing studies [21–24]. At the same time, as a viscoelastic material, indentation experiments show that the stress-strain response of porcine liver changes with the strain rate, and its change trend under different loading conditions is basically clear. Based on this principle, after grasping the elastic modulus corresponding to different gelatin concentrations, it is possible to further clarify the configuration method to make up for the difference in mechanical behavior between the two and appropriately increase or decrease the configuration concentration of gelatin.

In precision surgery research, loading behaviors that are difficult to perform in material experiments have emerged in some new applications. One example of this is the medical water jet, which can have a velocity range of tens of m/s to greater than 100 m/s. Therefore, the configuration of gelatin as an alternative requires more detailed research within the scope of current experiments, which will be an important work. In the current study, the mechanical behavior when simply using 10 wt.% or 20 wt.% gelatin instead of soft tissue needs further demonstration.

## Figures and Tables

**Table 1:** Six-order Prony series fitting result.

*n*	*s*_*i*_	*τ*_*i*_
1	0.1422	29.5
2	0.1087	2.341
3	0.1027	3.526
4	0.07156	0.05969
5	0.05719	0.389
6	0.04506	2.223

## Data Availability

The data used to support the findings of this study are available from the corresponding author upon request.
